# Feasibility Study for the Use of Gene Electrotransfer and Cell Electrofusion as a Single-Step Technique for the Generation of Activated Cancer Cell Vaccines

**DOI:** 10.1007/s00232-024-00320-5

**Published:** 2024-08-12

**Authors:** Marko Ušaj, Mojca Pavlin, Maša Kandušer

**Affiliations:** 1 Faculty of Health and Life Sciences, Department of Chemistry and Biomedical Sciences, Linnaeus University, 391 82 Kalmar, Sweden; 2 Faculty of Medicine, Institute of Biophysics, University of Ljubljana, Vrazov Trg 2, 1000 Ljubljana, Slovenia; 3 Faculty of Electrical Engineering, Group for Nano and Biotechnological Applications, University of Ljubljana, Tržaška 25, 1000 Ljubljana, Slovenia; 4 Institute for Pharmacy, Faculty of Pharmacy, University of Ljubljana, Aškerčeva 7, 1000 Ljubljana, Slovenia

**Keywords:** Electrofusion, Gene electrotransfer, Cancer cell vaccines, Cell membrane fluidity, Electroporation

## Abstract

Cell-based therapies hold great potential for cancer immunotherapy. This approach is based on manipulation of dendritic cells to activate immune system against specific cancer antigens. For the development of an effective cell vaccine platform, gene transfer, and cell fusion have been used for modification of dendritic or tumor cells to express immune (co)stimulatory signals and to load dendritic cells with tumor antigens. Both, gene transfer and cell fusion can be achieved by single technique, a cell membrane electroporation. The cell membrane exposed to external electric field becomes temporarily permeable, enabling introduction of genetic material, and also fusogenic, enabling the fusion of cells in the close contact. We tested the feasability of combining gene electrotransfer and electrofusion into a single-step technique and evaluated the effects of electroporation buffer, pulse parameters, and cell membrane fluidity for single or combined method of gene delivery or cell fusdion. We determined the percentage of fused cells expressing green fluorescence protein (GFP) in a murine cell model of melanoma B16F1, cell line used in our previous studies. Our results suggest that gene electrotransfer and cell electrofusion can be applied in a single step. The percentage of viable hybrid cells expressing GFP depends on electric pulse parameters and the composition of the electroporation buffer. Furthermore, our results suggest that cell membrane fluidity is not related to the efficiency of the gene electrotransfer and electrofusion. The protocol is compatible with microfluidic devices, however further optimization of electric pulse parameters and buffers is still needed.

**Graphical Abstract:**

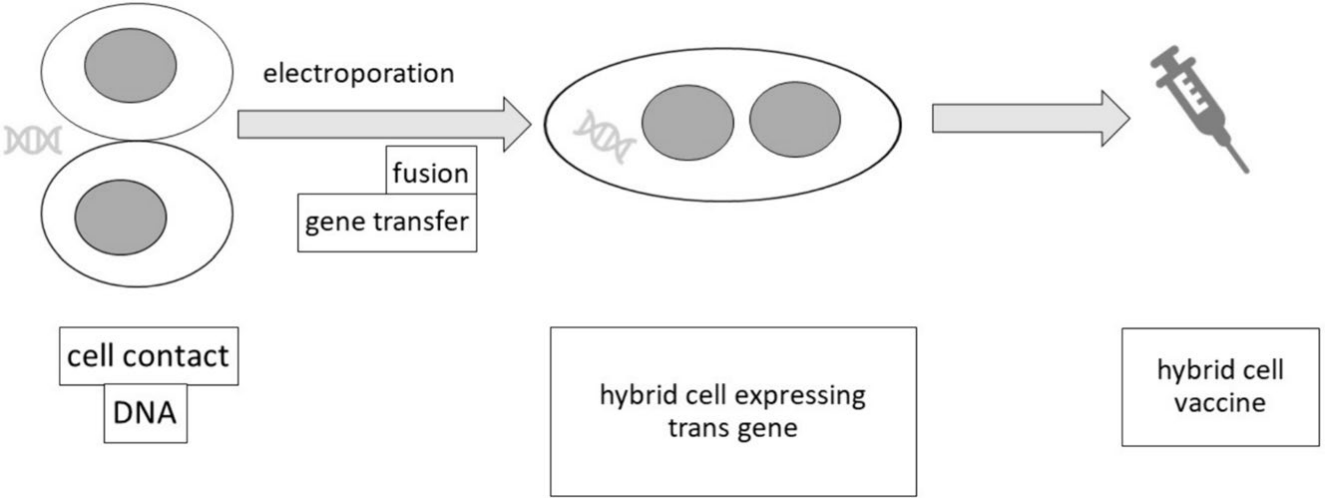

**Supplementary Information:**

The online version contains supplementary material available at 10.1007/s00232-024-00320-5.

## Introduction

Established cancer therapies frequently result in the development of drug resistance preventing successful treatment. To overcome such problems, an alternative strategy was proposed aiming to induce a potent anti-tumor response (Borghaei et al. 2009). The progress in the field of immunology provides new tools for development of prophylactic and therapeutic anti-cancer vaccines. DNA and whole cell vaccines have been designed and different clinical trials are underway. Among cell vaccines, dendritic cells (DC)-based vaccines are particularly promising for cancer therapy as DC can initiate, control, and regulate cellular and humoral immunity. To obtain tumor-specific immune response, selected or total tumor antigens are used to load DC. Loading of DC with tumor antigens can be accomplished by different approaches (Keenan and Jaffee 2012), including cell fusion of DCs and tumor cells (de Gruijl et al. 2008). The obtained hybrid cell couples the antigen-processing and immune-stimulatory potential of DCs with the whole antigenic spectrum of the tumor cell (Barbuto et al. 2004; Koido 2016; Rosenblatt et al. 2011).

However, DC-based vaccines have important limitations which include the maturation status of DCs (Lee 2011; Raaijmakers and Ansems 2018). These issues could be overcome by genetic manipulation as demonstrated in several studies (Tan et al. 2013). Genetic manipulation of DC can be used to stimulate IL-12 production (Shi et al. 2005; Suzuki et al. 2005; Tan et al. 2013) therefore some authors proposed that DC can be first genetically modified to promote DC maturation and then fused with tumor cells (Ogawa et al. 2004; Saxena et al. 2018).

Methods used for cell fusion and gene transfer are diverse; one of the possibilities for both, genetic manipulation and cell fusion is electroporation. Electroporation is a technique that uses high-voltage electric pulses to temporarily increase membrane permeability enabling the exchange of molecules between the cell interior and its surroundings (Canatella et al. 2001; Kotnik et al. 2019; Scuderi et al. 2022). Additionally, the permeable cell membrane has been proven to be fusogenic enabling the fusion of neighboring cells in the close contact (Zimmermann 1982). For effective application, one should choose the electric pulses to enable cell membrane permeabilization and at the same time to preserve cell viability. The effects of electric pulse parameters involved in the process have been studied extensively (Canatella et al. 2001; Rols and Teissié, 1998; Zimmermann 1982) and enabled the progress of electroporation which is currently used in several clinical trials for gene therapy and for the development of new medicinal products (Muraldharan and Boukany 2024). In gene therapy electroporation is suitable for electrotransfer of different kinds of nucleic acids, such as plasmid DNA, siRNA, and mRNA ex vivo or in vivo (Muralidharan et al. 2022; Muralidharan and Boukany 2024; Pavlin et al. 2024; Pavlin and Kanduser 2015).

The progress of electroporation from the bench to the bed site has been accompanied with the development of mathematical models. The mathematical models are valuable tools that provide explanation of the experimental results and elucidate the underlying mechanisms of electroporation (Kotnik et al. 2019; Pavlin and Miklavčič, 2008; Scuderi et al. 2022; Sözer et al. 2018). Previous (Pavlin et al. 2007; Sözer et al. 2018) and recent studies (Kotnik et al. 2019; Scuderi et al. 2022; Sözer et al. 2018) have shown that even for the simplest case, the transport of small molecules caused by electroporation, the existing models do not match experimental results (Muralidharan et al. 2021; Scuderi et al. 2022). Additionally, it has been accepted that the resealing of the cell membrane after electroporation is affected by biologic characteristics of the treated cell, especially actin and tubulin cytoskeleton (Muralidharan et al. 2021; Rosazza et al. 2011). Actin cytoskeleton also plays an active role in initial steps of gene electrotransfer of plasmid DNA (Rosazza et al. 2011), while tubulin network is probably involved in the transport of the plasmid in the cytosol (Rosazza et al. 2013). The role of tubulin network in cell fusion can be predicted also from the results of cell fusion in hypotonic buffer. Hypotonic treatment improves cell fusion yield but at the same time disrupts the tubulin network (Kandušer and Ušaj 2014). Even more, recent studies have shown that the transport of small molecules such as propidium iodide is affected by integrity of actin cytoskeleton (Muralidharan et al. 2021). The actin network is closely connected to the cell membrane, therefore cytoskeleton and cell membrane composition are important factors affecting cell membrane electroporation and the resealing process. Molecular dynamics studies suggest that electroporation affects the fluid-disordered membrane phases in local membrane regions of the lipid bilayer (Reigada 2014). The situation is even more complex in experiments performed on cell models. Some authors associate the disruption of actin network with the decrease in the membrane fluidity and the decrease of the resting transmembrane potential (Kim et al. 2020). In complex biologic membranes, the role of cell membrane fluidity in molecular transport or gene electrotransfer was not established unambiguously (Golzio et al. 2001; Kanduser et al. 2006, 2008; Rols et al. 1990). In living cell, the control. of membrane fluidity is important for cell homeostais (Levental et al. 2020), while the modulation of membrane fluidity enables cancer progression (Koundouros and Poulogiannis 2020). In some cases, cancer progression was associated with cell fusion (Lu and Kang 2009). In this context, we have indeed observed that higher electrofusion yield can be obtained with cancer cells compared to non-malignant ones (Ušaj et al. 2010) indicating that membrane fluidity might affect also electrofusion.

At the cellular level cell electrofusion and gene electrotransfer consists of several steps including cell membrane-based phenomenon and post-pulse processes that depend on cell signaling involved in cell membrane resealing. Cell membrane-related processes in gene electrotransfer are electropermeabilization, the interaction of the DNA with the cell membrane, complex formation, and translocation of the DNA across the membrane. The transport to the nucleus and gene expression are membrane independent (Golzio et al. 2002; Pavlin and Kanduser 2015) and relay on post-pulse cell responses. The main phases in cell electrofusion are formation of cell–cell contact, membrane permeabilization, fusion pore formation, membrane merging, cytoplasm mixing, and finally hybrid cell formation (Kozlovsky and Kozlov 2002). One of the crucial parameters for efficient electrotransfer or electrofusion is the induced transmembrane voltage (ITV) which was shown to present the limiting condition for the efficiency of both applications (Kotnik et al. 2019; Marjanovič et al. 2010; Pavlin et al. 2005; Ušaj et al. 2010). The current limitation of gene electrotransfer and electrofusion is relatively low efficiency (Chiu 2001; Kandušer and Ušaj 2014; Lu et al. 2015; Muralidharan et al. 2022; Rosazza et al. 2011; Skelley et al. 2009). Therefore, several microfluidic devices have been proposed to improve the output of the method (He et al. 2019; Tang et al. 2022). To improve the efficacy of cell electrofusion we can use different EP buffers, and methods for cell contact formation (Kandušer and Ušaj 2014). Cell electrofusion can reach between 5 and 10% depending on the approach used for cell contact establishment (Gabrijel et al. 2004; Kandušer and Ušaj 2014; Usaj et al. 2013). Electrofusion yield can be improved by application of nanosecond electric pulses (Rems et al. 2013; Wu et al. 2022) or by modified adherence method (Kandušer and Ušaj 2014). However, despite the low efficiency of electrofusion the method has been used in clinical applications for cancer treatment (Hawlina et al. 2022, 2023; Higano et al. 2010). We can expect that further improvements in the efficiency of electrofusion will facilitate its use in other areas of biomedical applications. The main direction is the development of microfluidic devices and compliant protocols. The advantage of multifluidic devices over bulk cell fusion is the controlled cell fusion achieved by specific cell pairing, better cell contact between fusion partners, and higher efficacy (He et al. 2019; Skelley et al. 2009). Recent studies demonstrate that also the efficacy of bulk gene electrotransfer (Marjanovič et al. 2010; Pavlin and Kanduser 2015) can be improved in microfluidic devices (Muralidharan et al. 2022). The developement of microfluidic devices and the adaptability of electroporation into the flow-through system (Muralidharan and Boukany 2024) demonstrate the need for further development of electroporation protocols that can be used in such systems.

Therefore, the present study aims to combine gene electrotransfer and cell electrofusion in a single-step procedure. We tested different EP buffers and pulse parameters.... in single step protocol. Additionally, we determined the effect of electroporation buffers and evaluated the effect of cell membrane fluidity in conditions affecting the efficacy of gene delivery and cell electrofusion. The application of one-step procedure for cell electrofusion and gene transfer has the potential application in DC-based cancer vaccines where fused cells could be simultaneously genetically modified for improved functionality. As a proof of concept, we combined conditions that enable transient cell membrane permeabilization required for gene electrotransfer and cell electrofusion of B16F1 cells that have been used as a simple cell model in our previous studies. Our results indicate the feasibility of the proposed methodology and the adaptability of the proposed protocol to microfluidic devices. Furthermore, we show that membrane fluidity cannot be related to conditions where we obtained optimal electrofusion or electrotransfer. Further studies are needed to optimize our proposed method and to integrate it with microfluidic devices.

## Materials and Methods

### Sample Preparation

Mouse melanoma cells (B16F1) were cultured in Dulbecco’s minimal essential medium (DMEM), supplemented with 10% fetal bovine serum (FBS), L-glutamine, and antibiotics crystacillin, and gentamicin and maintained in a humidified atmosphere at 37 °C and 5% CO_2_. Cells were grown in a 25-cm^2^ culture flask to obtain 70–80% confluence.

For dual-color microscopy, cells were incubated with blue 7-µM CMAC in isotonic KPB and red CMRA in Krebs Hepes buffer for 45 min at 37 °C in two separate 25-cm^2^ culture flasks. The staining solution was removed and cells were washed with culture media and maintained for an hour at 37 °C in the DMEM medium. In other experiments, we used unstained cells. In both cases, we prepared homogeneous cell suspension by 0.25% trypsin/EDTA solution. Cells were centrifuged for 5 min at 1000 rpm (180 × g) and resuspended in isotonic potassium phosphate buffer – KPB (10-mM K_2_HPO_4_/KH_2_PO_4_, 1-mM MgCl_2_) with 250-mM sucrose and osmolarity 262 mOsmol/kg. For dual-color electrofusion detection, we mixed blue and red cells in proportion 1:1, while for electrofusion detection via nuclear staining we have just resuspended the cells. Close cell–cell contacts were established by a modified adherence method (Ušaj and Kandušer 2015). A monolayer of spherical cells was obtained by placing 40-μl drop of cells in concentration 2 × 10^6^ cells/ml in each well of the 24-well plate (TPP, Switzerland). Cells were then incubated in 5% CO_2_ at 37 °C for twenty minutes to allow them to slightly attach to the surface of the culture dish, while maintaining a round shape. Before electroporation, cells were washed with isotonic buffer and then 350 μl of iso- or hypotonic electroporation buffer containing 40 µg/ml of plasmid coding for green fluorescent protein pEGFP-N1 was added. Plasmid was purified with Qiagen HiSpeed Plasmid Mega kit (Qiagen). Expression of encoded reporter gene (GFP) was used to analyze efficiency of gene electrotransfer. Optimal parameters for gene electrotransfer and cell electrofusion were determined in our previous studies (Kandušer and Ušaj 2014; Marjanovič et al. 2010; Pavlin and Kanduser 2015; Ušaj et al. 2010; Usaj and Kanduser 2012). Timing and changes in the cell radius due to the hypotonic cell swelling were recorded previously (Ušaj et al. 2009). For each treatment, we calculated induced transmembrane potential, taking into account swelling of cells exposed to hypotonic buffer. The cells changed their size from radius* r* = 8.1 ± 1.1 µm in isotonic buffer to *r* = 9.3 ± 1.8 µm in hypotonic buffer (Ušaj et al. 2010).

The induced transmembrane voltage (ITV) on a spherical cell can be obtained from Schwan equation (Pavlin et al. 2005; Zimmermann 1982).

1 $$ITV=1.5rEcos\varphi,$$

where *r* is the radius of the cell, *E* is the strength of the external electric field, and *φ* is the angle between the direction of the external electric field and the normal from the center of the cell to the point of interest on the cell surface. From this, the maximal *induced transmembrane voltage ITV*_*max*_* can be* calculated as described earlier (Usaj and Kanduser 2012):

2 $$ITVmax=1.5 r E.$$

### Electroporation and Cell Electrofusion

Cells were electroporated in different iso- or hypotonic buffers (Table 1). In the protocols where we used hypotonic treatment cells were maintained in the hypotonic buffer for two minutes to reach their maximal volume and then electric pulses were delivered. Electric pulse parameters were applied as a train of 8 × 100 μs electric pulses or 4 × 200 μs at repetition frequency 1 Hz and amplitude 1.4 kV/cm and 1.6 kV/cm. Besides, we tested the combination of high-voltage (HV) and low-voltage (LV) electric pulses HV + LV with the HV 4 × 200 µs, 1.4 kV/cm, 1 Hz, and LV 1 × 100 ms, 0.014 kV/cm. The composition and osmolarity of electroporation buffers used for gene electrotransfer and cell fusion and electric pulse parameters are presented in Table 1. As electroporation buffers, we used isotonic KPB (iso KPB), isotonic KPB with Ca^2+^ ions (0.1-mM calcium acetate), or hypotonic KPB containing 75-mM sucrose with osmolarity 93 mOsmol/kg with conductivity 1.67 mS/cm and pH 7.2. Additionally, we tested, the commercially available Eppendorf® hypo-osmolar buffer (Epphypo; cat. number 36205-60) and the iso-osmolar buffer (Eppiso; cat. number 36205-62).

**Table 1:** Different electroporation buffers and electric pulse parameters used in gene electrotransfer/ electrofusion

Electroporation buffer	Buffer osmolarity	Electric pulse parameters
Eppiso/Epphypo Eppendorf® containing 0.5-mM Mg acetate, 0.1-mM Ca acetate	iso/hypo cat. number 36205–60/ 36,205–62	HV: 4 × 200 µs; 8 × 100 µs
Iso/hypo KPB 10-mM K_2_HPO_4_/KH_2_PO_4_, 1-mM MgCl_2_ 250/75-mM sucrose	iso/hypo 262 /93 mOsmol/kg	HV: 4 × 200 µs; 8 × 100 µs; HV + LV: 4 × 200 µs + 100 ms
Iso KPB + Ca 10-mM K_2_HPO_4_/KH_2_PO_4_, 1-mM MgCl_2_ 250-mM sucrose 0.1-mM Ca acetate	iso 262 mOsmol/kg	HV: 4 × 200 µs

For electroporation we used two parallel wire electrodes (Pt/Ir = 90/10) with five mm gap. No pulses were applied in the control treatment. After pulse delivery, the cells were left undisturbed for ten minutes for cell fusion to take place. Fusion yield was determined 30 min and 24 h after electroporation by dual-color fluorescence microscopy or by determination of polynucleated cells. Complete culture media was added 10 min after electroporation and images were acquired by inverted fluorescence microscope Zeiss Axiovert 200 (Zeiss, Germany) at 20 × objective magnification. Brightfield and fluorescence images of blue CMAC (excitation/emission 353 nm⁄466 nm, Chroma, USA), red CMRA (excitation/emission 548 nm/576 nm, Chroma, USA), and green GFP-positive cells (excitation/emission 488 nm/ 507 nm, Chroma, USA) were acquired. Alternatively, cell nuclei were stained with nucleic acid stain Hoechst 33,342 (excitation/emission 361 nm/497 nm) for 15 min at 37 °C and washed with cell culture media.

### Determination of Electrofusion Yield and Efficacy of Gene Electrotransfer

The fusion yield and gene transfer efficacy were determined in multi-channel images. The channels were brightfield and the fluorescence channels of blue CMAC, red CMRA, and green (GFP). Multiple images (bright field, blue, red, and green fluorescence) were acquired from five randomly chosen fields for each sample using cooled CCD video camera VisiCam 1280 (Visitron, Germany) and PC software MetaMorph 5.0. (Molecular Devices, USA). Images were merged into multi-channel image in the image processing software ImageJ (NIH Image, USA). Alternatively, bright field and Hoechst fluorescence images were used to determine multinucleated cells resulting from cell fusion. The cells were manually counted and the electrofusion yield was calculated as a percentage of dual-color cells (i.e., cells with red and blue fluorescence) (Ušaj and Kandušer 2015) or multinucleated cells (i.e., presence of two or more nuclei in an individual cell) (Trontelj et al. 2008; Usaj et al. 2013). Hoechst is a membrane-permeable dye suitable for live cell imaging. With the combination of brightfield image and Hoechst fluorescence, we can readily determine the cells with more than one nucleus per cytoplasm. Since a certain amount of polynucleated cells is always present in the unsynchronized cell culture we also determined the number of the polynucleated cells in the control sample and subtracted them from the number of polynucleated cells in the treated sample (Eq. 3).

3 $$\% \,poly\,nucleated\,cells\, = \,\frac{{N_{pt} - N_{pc} }}{N\,all\,cells}\, \times \,100$$

where N_pc_ is the number of polynucleated cells in the control, N_pt_ is the number of polynucleated cells in the treated sample, and N_all cells_ represent the number of all cells per given sample.

Besides cell fusion yield, we determined the efficacy of gene electrotransfer as the percentage of GFP-positive cells. Uniquely for this study, the percentage of transfected fused cells expressing GFP (GFP-hybrids) was determined by detecting green staining in polynucleated cells (Eq. 4).

4 $$\% GFP-hybrids=\frac{{N}_{GFP-hybrid}}{{N }_{all cells}}\times 100,$$

where N_GFP-hybrids_ represent the number of GFP-positive fused cells and N_all cells_ represent the number of all cells per given sample.

### Measurements of Cell Membrane Fluidity

Membrane fluidity was determined in two cell lines, B16F1 and CHO-K1, used in our previous studies of gene electrotransfer and cell electrofusion. Cells were seeded at appropriate cell density. On the day of the experiment, we prepared aliquots of cells in suspension and centrifuged them for 5 min at 4 °C at 1000 rpm (180 × g). We kept pellets at 4 °C. Cell pellet was resuspended to obtain a concentration of 10^5^ cells/ml, which was stained for 5 min at 37 °C with 1-µM TMA-DPH in isotonic KPB buffer. Stained cells were then centrifuged again and cell pellets were re-suspended in iso- or hypotonic KPB buffer. Two minutes later, cell membrane anisotropy was determined as described in (Rols et al. 1990) by polarized fluorescence intensity measurements using FP-6300 (Jasco, USA) equipped with a manual UV/VIS polarizer (FDP-223).

The fluorescence anisotropy r is calculated as follows:

5 $$r = \frac{Ivv - g\, \times IvH}{{Ivv + 2g \times \,IvH}}\quad,$$

where *g* is the correction factor of the instrument, *Ivv* is the fluorescence emission intensity detected with the parallel orientation of the polarizer, and *IvH* is the fluorescence emission intensity with the perpendicular orientation of the polarizer to polarization of the excitation light. The fluorescence anisotropy *r* is inversely proportional to cell membrane fluidity.

### Chemicals and Reagents

The chemicals were purchased from Sigma (Sigma-Aldrich Chemie GmbH, Germany). Antibiotics (crystacillin and gentamicin) from Pliva (Pliva d.o.o, Croatia). Cell trackers blue CMAC and red CMRA as well as TMA-DPH were from Molecular probes (Invitrogen, USA) and pEGFP from Clontech. Hoechst 33,341 dye was from Sigma (Sigma-Aldrich).

## Results

In Table 2, we present the efficiencies of cell electrofusion, gene electrotransfer, and feasibility of generation of fused cells expressing GFP (GFP-hybrids) in single-step procedure. Experiments were performed in commercially available iso- and hypo-osmolar Eppendorf buffer (Epp iso, Epp hypo). We used two sets of electroporation parameters, 4 × 200 µs electric pulses (previously determined to be optimal for electro gene transfer in this cell line) and 8 × 100 µs electric pulses previously used for electrofusion of B16F1 cells. The cells exposed to hypotonic buffer swell and increase their size, therefore electric pulse amplitudes have to be adjusted to obtain the comparable induced transmembrane potential for all treatments. The percentage of fused cells increases in hypotonic conditions. The percentage of gene electrotransfer (% GFP-positive cells) and the electrofusion yield (% ECF) was higher in the hypotonic buffer and lower in the isotonic buffer when we compared commercially available iso- and hypo-osmolar Eppendorf buffer. In these buffers, we did not obtain any GFP-hybrids.

**Table 2:** Percentage of cell electrofusion (ECF), gene electrotransfer (GFP), and intersection of both (GFP-hybrids) for two sets of electric pulse parameters (4 × 200 µs and 8 × 100 µs), where the maximal induced transmembrane voltage ITV_ma**x**_ = 1.7 V

Eppendorf® buffer used, pulse number, pulse duration (µs)	E—pulse amplitude [kV/cm]*	ECF (%)	GFP (%)	GFP-hybrids (%)	ITV_ma**x**_ [V]
Epp hypotonic- 4 × 200	1.2	6	7	0	1.7
Epp hypotonic- 8 × 100	1.2	8	7	0	1.7
Epp isotonic- 4 × 200	1.4	2	4	0	1.7
Epp isotonic- 8 × 100	1.4	1	3	0	1.7

The electric pulse amplitudes were *E*_*iso*_ = 1.4 kV/cm in isotonic and *E*_*hypo*_ = 1.2 kV/cm in hypotonic Eppendorf® electroporation buffers containing [Mg acetate] = 0.5 mM, [Ca acetate] = 0.1 mM. Electrofusion yield (determined by dual-color assay) and gene electrotransfer efficiency were assessed 24 h after treatment

*at selected pulse amplitude *ITV*_*max*_ was always 1.7 V

**Fig. 1:**
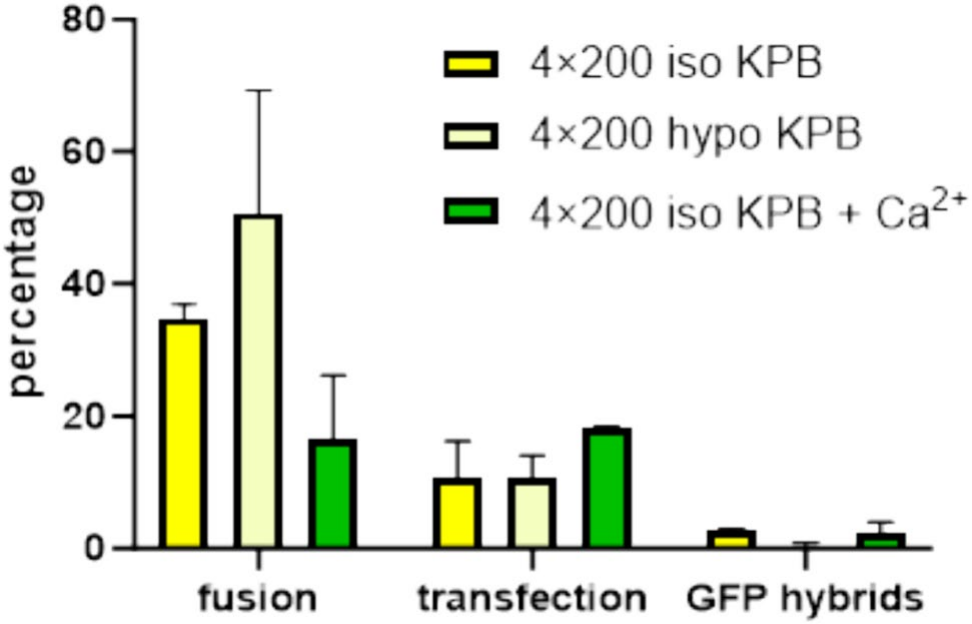
Effect of different KPB electroporation media on the percentage of cell electrofusion, GFP gene electrotransfer (transfection) and electrofused cells expressing GFP (GFP– hybrids) assessed 24 h after electric pulses. Electric pulse parameters were 4 × 200 µs delivered at 1 Hz. Electric field amplitude [E] in isotonic buffer was 1.4 kV/cm and 1.2 kV/cm in hypotonic buffer preserving ITV_max_ at the same value of 1.7 V. Cell fusion was estimated by the presence of multinucleated cells detected by Hoechst staining 10 min after application of electric pulses. Bars are means of two independent experiments ± standard deviation. Approximately 100 cells were counted in each image and at least 5 images were analyzed per each treatment. Differences among treatments were analyzed by 2-way ANOVA and compared by Bonferroni’s multiple comparison test. The differences among the treatments were not statistically significant

For further experiments, we used our own KPB buffer alone or with the addition of calcium ions for improved gene electrotransfer (Haberl et al. 2010, 2013). In Fig. 1, we present the percentages of cell electrofusion (hybrids), gene electrotransfer (transfection), and GFP-hybrids in different KPB buffers: isotonic KPB (iso KPB), hypotonic KPB (hypo KPB) and isotonic KPB with addition of [Ca^2+^] = 0.1 mM (iso KPB + Ca). As expected the highest fusion yield was obtained in hypotonic buffer. The percentage of cells expressing GFP was slightly higher in isotonic buffer containing Ca^2++^ ions. The percentage of GFP-hybrids was higher in isotonic buffers (2%) compared to hypotonic buffers (0.4%).

**Fig. 2:**
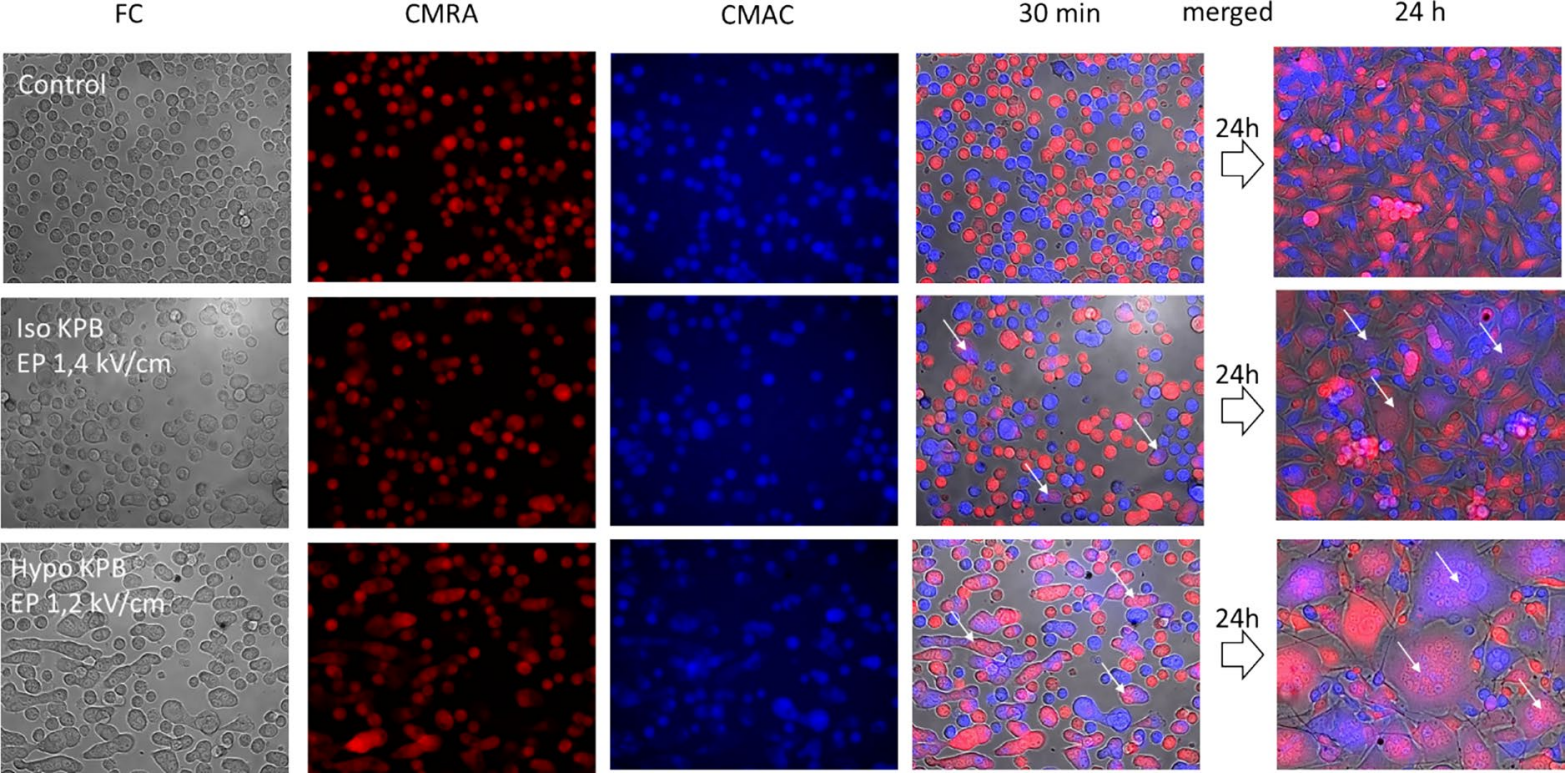
Images of B16F1 cells after electrofusion in isotonic (iso KPB) and hypotonic (hypo KPB) buffer were recorded at 30 min (separate channels and merged images) and 24 h (merged image) after electric pulse application (4 × 200 µs delivered at 1 Hz). Electric pulse amplitudes were 1.2 kV/cm for hypotonic, 1.4 kV/cm for isotonic buffer, and 0 kV/cm for control. Cells were stained with red (CMRA) and blue (CMAC) cell tracker. Successfully fused cells are violet in color (the merge contains red and blue cell tracer in their cytoplasm) and some of them are marked with white arrows in merged images

In Fig. 2, we present the time course of cell electrofusion in hypotonic and isotonic buffer with respect to control treatment (first row—no electric pulse and hypotonic treatment). Panels on the left represent cells 30 min after while the panel on the right shows results 24 h after the electric pulse application. The electric pulse amplitude was adapted to electroporation media (i.e. isotonic in the middle or hypotonic at the bottom of the figure) to preserve constant induced transmembrane voltage.

**Fig. 3:**
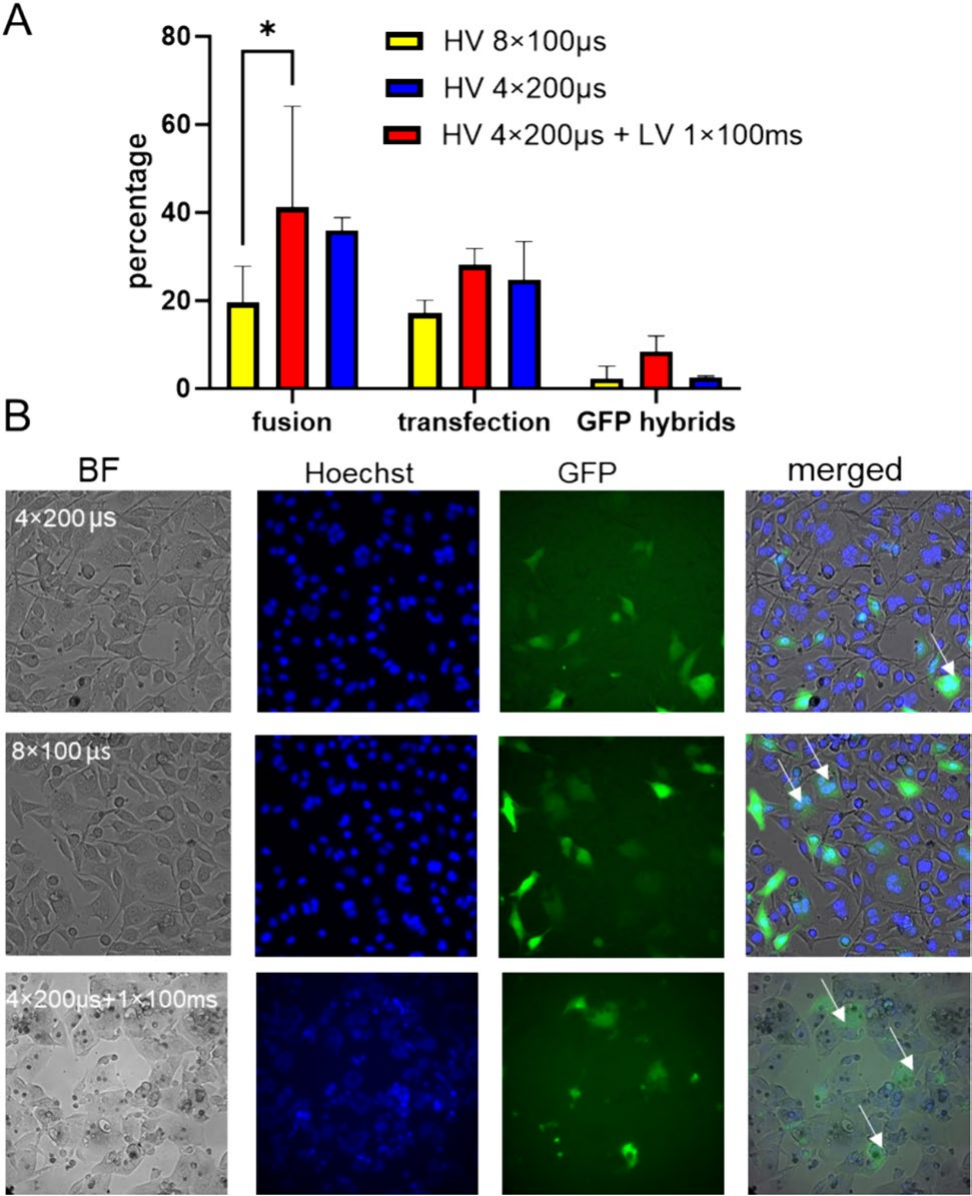
The effect of pulse parameters on the percentage of cell electrofusion (hybrids), gene expression (GFP), and fused cells expressing GFP (GFP-hybrids) in isotonic KPB buffer. We compared 4 × 200 µs and 8 × 100 µs (HV) pulses and a combination of HV (4 × 200 µs) and one LV_55_ pulse (100 ms) (HV + LV). Electric field amplitude E for HV was 1.4 kV/cm and 0.11 kV/cm for LV_55_. In the panel (A), we present the percentages of cell electrofusion, gene electrotransfer (GFP), and GFP-hybrids. Bars are means of 3 independent experiments ± standard deviation. The higher number of hybrids is obtained when we used pulses combination of HV and LV pulses compared with HV (4 × 200 µs) pulses alone; however, the difference was not statistically significant. In the panel (B) we present three-channel micrographs of the treated cells (brightfield, cell nuclei stained with Hoechst-blue, cells expressing GFP-green) and merged images. Cell electrofusion and GFP expression were detected 24 h after electric pulse application. Images were acquired at 20 × objective magnification. Multinucleated cells expressing GFP (GFP-hybrids) are indicated with arrows. Approximately, 100 cells were counted in each image and at least 5 images were analyzed per each treatment. Differences among treatments were analyzed by 2-way ANOVA and compared by Bonferroni’s multiple comparison test (*p = 0.0492)

To increase the percentage of GFP-hybrid cells we have tested the combination of high-voltage (HV) and low-voltage (LV) pulses in iso KPB, because the percentage of GFP-hybrids was higher in isotonic buffers. In Fig. 3, we present the percentages of cell electrofusion (fusion), GFP gene electrotransfer (transfection), and GFP-hybrid cells for different electric pulse parameters. The highest percentage of GFP-hybrid cells was obtained when we used the combination of high-voltage and low-voltage pulses reaching 9% of total cells used in experiment (Fig. 3A). In tree channel micrographs, we present treated cells. The combination of high-voltage and low-voltage pulses resulted in large polynucleated cells with dim fluorescence of GFP in GFP-hybrids (Fig. 3B). Polynucleated cells are the result of random unspecific cell pairing which is a current drawback of existing cell fusion methods. Dim fluorescence of GFP in GFP-hybrids is most likely the consequence of large cytoplasmic volume and high number of nuclei in a single cytoplasm which are interrupting normal cell function required for proper expression of GFP. The situation might be specific for artificially obtained polynucleated cells as such situation was not observed in naturally polynucleated muscle cells (Velayuthan et al. 2023).

In supplementary Figure S1, we present also the fraction of fused cells expressing GFP where we noted that 24% of all hybrids have expressed GFP when we used a combination of high-voltage and low-voltage pulses.

To determine the role of biophysical properties of the cell membrane on the proposed one-step procedure we investigated the effect of cell membrane fluidity on gene electrotransfer and cell electrofusion. We performed polarization studies that measured the steady-state anisotropy (r) using fluorescent probe 1,6-diphenyl-1,3,5-hexatriene; TMA- DPH that mimics the molecular movements in the membranes of the analyzed sample. Since r refers to the rigidity and fluidity refers to the viscosity of lipid layers, the fluidity index is the inverse value of r-anisotropy (i.e. 1/r).

**Fig. 4:**
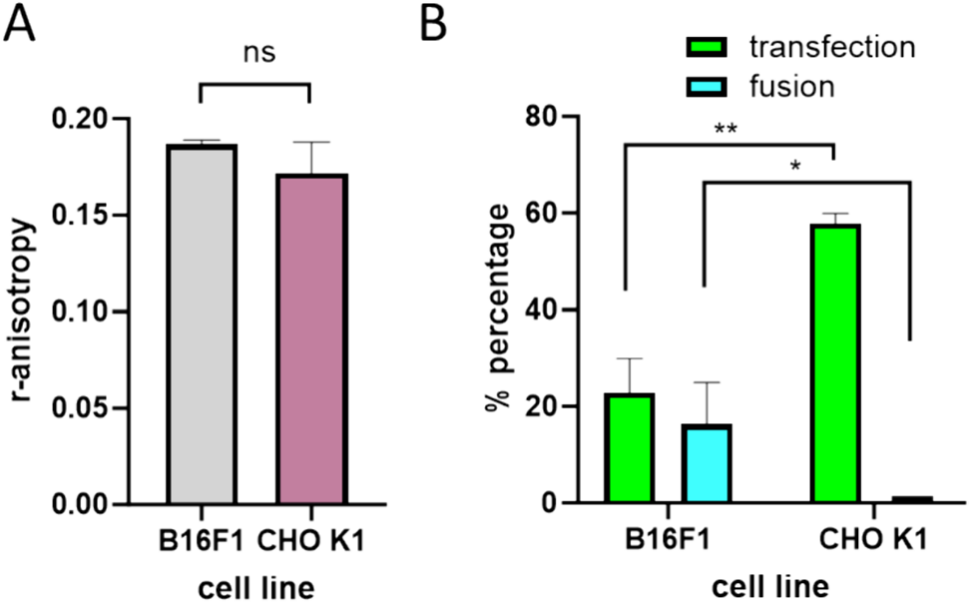
Comparison of r-anisotropy and efficiency of electrotranfection and electrofusion in two cell lines (B16F1 and CHO-K1) that significantly differ in their GFP gene electrotransfer (transfection) (Marjanovič et al. 2010) and cell electrofusion (fusion) (Usaj and Kanduser 2012). In the panel (A) r-anisotropy is shown, fluidity is the inverse value of r (i.e., 1/r-anisotropy). In the panel (B) we present gene electrotransfer and cell electrofusion for the electroporation pulse parameters that have the same ITV_max_ = 1.7 V in KPB isotonic buffer. The data represent the mean ± standard deviation of three independent experiments (*p = 0.0298, **p = 0.0032)) as determined by *t* test

To analyze the effect of cell membrane fluidity on gene electrotransfer, cell electrofusion, and on one-step procedure, we measured r-anisotropy of the cell line B16F1 and compared it with CHO-K1 cell line that differs from B16F1 in gene electrotransfer and cell electrofusion efficiency. In Fig. 4, we present the results of r-anisotropy for the two cell lines and the results for gene electrotransfer (Marjanovič et al. 2010) and cell electrofusion (Usaj and Kanduser 2012) obtained in our previous studies. Summarized results (Fig. 4) show no relation between the membrane fluidity and electrogene transfer or cell electrofusion. Even if a comparison in the percentage of cells expressing GFP between CHO (23%) and B16F1 (58%) could be potentially related to the observed differences in r-anisotropy (0.171 vs 0.182; *p* = 0.03, CHO, and B16F1, respectively) it is interesting to note that we did not obtain any cell electrofusion of CHO cells compared to the 16% fused B16F1 cells already in isotonic conditions. Hypotonic buffer significantly increased electrofusion of both cell lines, but it does not affect r-anisotropy (supplementary Table 1). Interestingly, however, when we related r-anisotropy with the efficiency of generation of GFP-hybrids for B16F1 in hypotonic and isotonic buffers we observed a 2.7% of GFP-hybrids in isotonic buffer and only 0.4% in hypotonic buffer (Fig. 1).

All the r-anisotropy measurements were performed at room temperature (22 °C), while as additional control r-anisotropy samples were also measured in isotonic buffer at 4 °C (supplementary Table 1) where cell membrane fluidity was decreased as demonstrated also in our previous study (Kanduser et al. 2008). The measurements effectively validated the method used in this study for membrane fluidity measurements.

## Discussion

The progress in immunology has led to breakthroughs in the development of prophylactic and therapeutic anti-cancer vaccines (Borghaei et al. 2009). For the development of effective vaccine platforms, different approaches have been used (Fajardo-Moser et al. 2008; Keenan and Jaffee 2012; Laureano et al. 2022). The gene electrotransfer and cell electrofusion are versatile techniques that enable genetic manipulation and cell fusion (Lin et al. 2022; Patente et al. 2019; Perez and De Palma 2019; Saxena et al. 2018), holding great potential for different vaccine platforms. We developed a one-step electroporation protocol enabling simultaneous cell fusion and genetic manipulation. To understand the effect of cell membrane fluidity on the protocol, we also evaluated the r-anisotropy in conditions that affect gene electrotransfer and cell electrofusion.

The feasibility of the single-step technique was evaluated in B16F1 melanoma cancer cell line which was characterized in our past studies (Kandušer et al. 2009; Marjanovič et al. 2010; Pavlin et al. 2010; Ušaj et al. 2010; Usaj and Kanduser 2012) and it is frequently used in preclinical studies of DC-based cancer vaccines (Edele et al. 2014; Gordy et al. 2016; Mac Keon et al. 2015; Wang et al. 1998). We successfully developed a protocol that combines electrofusion and gene electrotransfer in a single procedure. We tested different electroporation media and electric pulse parameters previously optimized separately for electropermeabilization, gene electrotransfer (Marjanovič et al. 2010; Kandušer et al. 2009; Pavlin et al. 2010; Haberl et al. 2013), and cell electrofusion (Trontelj et al. 2008; Ušaj et al. 2010; Usaj and Kanduser 2012) and evaluated the effectiveness of selected parameters for the one-step protocol. The efficiency of the protocol was evaluated as the yield of GFP expressing hybrids (GFP-hybrids), which depended on the electroporation buffer (Table 1, Figs. 1, 2) and on electric pulse parameters (Fig. 3). Our results demonstrate that the hypotonic treatment which is the best choice for cell electrofusion (Fig. 2) was not the best option for the one-step procedure (Fig. 1), therefore we tested additional electric pulse parameters using isotonic buffer. The highest percentage of GFP-hybrids (9%) was obtained with the combination of high-voltage (HV) and low-voltage (LV) electric pulses (Fig. 3). This is in agreement with our previous work where we have already shown that the combination of HV and LV pulses increased the efficiency of gene electrotransfer in CHO cells at suboptimal DNA concentrations (Kanduser et al. 2009; Pavlin et al. 2010). The results indicate that for the generation of transfected hybrids, it is also important to provide electrophoretic low-voltage pulses to enable efficient contact between DNA and the cell membrane which is crucial for efficient gene electrotransfer (Golzio et al. 2002; Haberl et al. 2013; Kandušer et al. 2009; Pavlin et al. 2010). In the present study, we used optimal plasmid concentration for gene electrotransfer; however, dense cell suspension and tight cell contacts required for cell fusion might hinder plasmid availability at the cell membrane level. The highest yield of GFP-hybrids (Fig. 3, Fig. S1) can be therefore explained with the improved DNA-membrane contact and higher degree of GFP expression (Fig. S1) like in conditions of suboptimal plasmid concentration. Interestingly, we observed that the efficiency of electrofusion increased on average by 15% when we used fewer pulses with longer duration (4 × 200 µs compared to 8 × 100 µs), with a total duration of pulses being the same (i..e. 800 µs) or combination of high- and low-voltage pulses. The difference was statistically significant only when we compare 8 × 100 µs pulses with 4 × 200 µs + 1 × 100 ms (*p* = 0.05). This finding may pave a new way into optimization of cell electrofusion itself, especially for low fusogenic cell lines (Salomskaitė-Davalgienė et al. 2009). To combine gene electrotransfer and cell electrofusion we had to find a compromise; we have to omit fetal bovine serum that is typically added after electroporation in gene electrotransfer protocols (Golzio et al. 2002; Kandušer et al. 2009; Pavlin et al. 2010) because such intervention has negative impact for electrofusion where cells have to be left undisturbed. The selected protocols (Figs. 1, 3) otherwise enabled efficient electrotransfection and high fusion yield as shown in our previous studies (Kandušer et al. 2009; Marjanovič et al. 2010; Pavlin et al. 2010; Pavlin and Kanduser 2015; Usaj and Kanduser 2012) where less effective in the combined method. The optimal experimental conditions for these two separate processes are not trivial to achieve while combining them presents additional challenges. In the majority of common bulk cell fusion protocols, we face the problem of unspecific cell pairing and formation of large polynucleated hybrids. We show here that such hybrids have a limited capacity to express GFP (Fig. 3B); however, this challenge can be solved by specific cell fusion in microfluidic devices as discussed later. Our results indicate that one-step technique is possible with further optimization of electric field parameters, electroporation buffers, specific cell pairing, and better understanding of other experimental and biophysical parameters affecting both processes.

Besides external factors, we investigated also the effect of cell membrane fluidity which might affect the one-step protocol and the generation of GFP-hybrids. Cell membrane fluidity could be involved in complex or contact formation required for gene electrotransfer or electrofusion, respectively (Golzio et al. 2002; Kandušer and Ušaj 2014; Kozlovsky and Kozlov 2002; Pavlin and Kanduser 2015). Additionally, EP and cell fusion depend on the lipid composition of the cell membrane (Gabriel and Teissie 1994; Kanduser et al. 2019; Kotnik et al. 2019; Kozlovsky and Kozlov 2002; Maccarrone et al. 1995; Markelc et al. 2012) and we observed significantly higher electrofusion yield in melanoma cell line B16F1 compared to non-cancer cell line CHOK1 (Fig. 4B) which could be related to changes in membrane fluidity observed in malignant transformation (Koundouros and Poulogiannis 2020). However, the fluidity in both cells lines was very similar (Fig. 4A) (Usaj and Kanduser 2012) demonstrating that fluidity of the membrane is not a crucial parameter for electroporation, gene electrotransfer or electrofusion process. This finding is not supporting the results of molecular dynamics simulations of pure lipid bilayers.

The molecular dynamics simulations of pure lipid bilayers have shown that hydrophobic pores are formed mainly in small disordered membrane domains which implies that fluidity is an important parameter (Reigada 2014; Shigematsu et al. 2014), while also older experimental results in cells do not support molecular dynamic findings (Golzio et al. 2001; Kanduser et al. 2006, 2008; Rols et al. 1990). To clarify the role of cell membrane fluidity in electrotransfection, we summarized the results in conditions that result in different percentage of gene electrotransfer (Marjanovič et al. 2010) and related them to membrane fluidity (Fig. 4, Supplementary Table S1; Fig. S2). Our results demonstrate clearly that membrane fluidity (Fig. 4 A) could not be related to the differences in gene electrotransfer obtained with different cell lines or EP protocols (Figs. 3, 4 B; Fig S2, Fig S3), as the % of electrotransfection was significantly higher in CHO cells compared to B16F1 cells, while no differences in membrane fluidity were detected. Similar results were obtained for cell electrofusion. Altogether our results show that electroporation as defined in simplified lipid models is therefore not relevant neither for gene electrotransfer nor for electrofusion. This observation is further supported by our previous results (Pavlin et al. 2007; Pavlin and Miklavčič, 2008) demonstrating that the stability of long-lived pores and their time constant of resealing do not behave as pores in purely lipid systems that have very short resealing time even for smaller molecules, which was also confirmed by a very recent study (Muralidharan et al. 2021) demonstrating that even much simpler transport of small molecules is not controlled only by cell membrane electropermeabilization. Our results are therefore also in line with recent studies and models showing that that membrane complexity has to be taken into account to match experimental observations (Kotnik et al. 2019; Scuderi et al. 2022; Sözer et al. 2018). Furthermore, the actin network which was previously related to the efficacy of gene electrotransfer of plasmid DNA (Rosazza et al. 2011) was recently related also to the transport of small molecules (Muralidharan et al. 2021). The fact that actin cytoskeleton affects cell membrane fluidity (Dinic et al. 2013; Kim et al. 2020) and is also involved in gene electrotransfer (Rosazza et al. 2011) and transport of small molecules (Muralidharan et al. 2021) indicate that many interconnected biologic factors affect electroporation (Muralidharan et al. 2022). Thus, our experimental data are in agreement with the studies showing that gene electrotransfer or electrofusion cannot be fully predicted by existing models and deeper understanding of biologic factors such as cytoskeleton needs to be taken into account (Scuderi et al. 2022).

The one-step protocol presented here is a proof of a concept; we can combine electrofusion and gene electrotransfer in a single step. From the previous studies, we know that the efficacy of gene electrotransfer depends on the cell line and the state of the treated cells (in suspension vs plated cells), days in the culture, density of cell culture, trypsinization, and cell cycle (Marjanovič et al. 2010; Pavlin and Kanduser 2015). The efficiency of gene electrotransfer ranges from 25 to 40% of transfected cells (Pavlin and Kanduser 2015) and we would like to stress that the reported percentage is also dependent on the conditions of image analysis for both gene electrotransfer (Muralidharan et al. 2022) and cell electrofusion (Gabrijel et al. 2004). The efficiency of bulk cell fusion obtained with the most commonly used chemical agent polyethylene glycol (PEG) reaches around 10% (Chang et al. 2023) which is in the range of GFP-hybrids obtained in our study (9%). The fusion yield obtained by electroporation depends on two factors, cell membrane permeabilization and the method used for establishment of cell contacts. Initial % of bulk fusion of cells aligned with DEF was around 5% (Gabrijel et al. 2004; Kandušer and Ušaj 2014) and can be improved with different methods (Kandušer and Ušaj 2014; Rems et al. 2013; Wu et al. 2022). However, the main limitation of bulk cell fusion remains unspecific cell pairing (Kandušer and Ušaj 2014) responsable for the formation of large multinucleated cells. Many nuclei in a cytoplasm can negatively affect expression of protein encoded in the plasmid resulting in low % of GFP-hybrids (Fig. 3). Those limitations can be overcome by technological advances in microfluidics (He et al. 2019; Kandušer and Ušaj 2014; Skelley et al. 2009; Wu et al. 2022). The best results for properly paired and fused cells were obtained in the device based on a dense array of hydrodynamic traps (Skelley et al. 2009). Similar device was very effective also for gene electrotransfer. The main advantage of our proposed one-step protocol is compatibility with microfluidic devices which enable high yield of either fusion (Skelley et al. 2009) or gene transfer (Muralidharan et al. 2022).

**Fig. 5:**
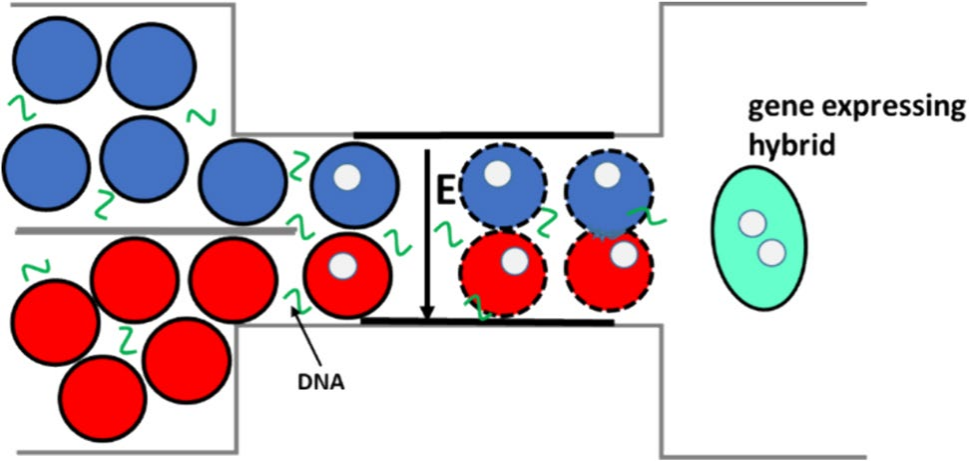
The schematic representation of the implementation of the single-step protocol (demonstrated here in our study) in existing microfluidic devices developed for cell fusion (Skelley et al. 2009) or electro gene transfer (Muralidharan et al. 2022)

Based on this information we believe that the existing results of 9% GFP-hybrids is a good starting point that can be optimized by specific cell fusion in microfluidic devices. We belive that the device developed by Muralidharan (Muralidharan et al. 2022) for gene electrotransfer and Skelly for cell electrofusion (Skelley et al. 2009) could be easily adapted also for our one-step protocol. From this prospective, our one-step protocol presents an advantage and its implementation to microfluidic devices is shown schematically in Fig. 5. We expect that in microfluidic device cell fusion and gene electrotransfer will increase due to improved viability, specific cell pairing, and higher gene expression, affecting also the final yield of GFP-hybrids. In such microfluidic device the limited availability of the plasmid and unspecific cell fusion which is producing large multinucleated cells would be resolved.

With such implementation of gene electrotransfer and cell electrofusion in microfluidic device, the protocol has the potential for the development of therapeutic platforms for cancer cell vaccines to produce genetically modified hybrids expressing genes encoding adjuvants or cytokines required to improve vaccine efficacy.

In conclusion, we demonstrated that gene electrotransfer and cell electrofusion can be applied as a one-step protocol. The percentage of viable fused cells expressing GFP mainly depends on electric pulse parameters and electroporation buffers. For the selected electroporation buffers that improved gene electrotransfer, cell electrofusion, and/or formation of GFP-hybrids we had not observed alterations in cell membrane fluidity. Therefore, our data provide further evidence that more complex molecular and biophysical cell membrane models are needed considering the high concentration of membrane proteins and the interactions of the cell membrane with the cell cortex and cytoskeleton to bridge the gap between current models and experimental results.

## Supplementary Information

Below is the link to the electronic supplementary material.

Supplementary file1 (DOCX 197 kb)

## Data Availability

No datasets were generated or analyzed during the current study.

## References

[CR1] Barbuto JAM, Ensina LFC, Neves AR, Bergami-Santos PC, Leite KRM, Marques R, Costa F, Martins SC, Camara-Lopes LH, Buzaid AC (2004) Dendritic cell?tumor cell hybrid vaccination for metastatic cancer. Cancer Immunol Immunother 53:1111–1118. 10.1007/s00262-004-0551-7 15185011 10.1007/s00262-004-0551-7 PMC11032787

[CR2] Borghaei H, Smith MR, Campbell KS (2009) Immunotherapy of cancer. Eur J Pharmacol 625:41–54. 10.1016/j.ejphar.2009.09.067 19837059 10.1016/j.ejphar.2009.09.067 PMC2783916

[CR3] Canatella PJ, Karr JF, Petros JA, Prausnitz MR (2001) Quantitative study of electroporation-mediated molecular uptake and cell viability. Biophys J 80:755–764 11159443 10.1016/S0006-3495(01)76055-9 PMC1301274

[CR4] Chang CY, Tai JA, Sakaguchi Y, Nishikawa T, Hirayama Y, Yamashita K (2023) Enhancement of polyethylene glycol-cell fusion efficiency by novel application of transient pressure using a jet injector. FEBS Open Bio 13:478–489. 10.1002/2211-5463.13557 36651034 10.1002/2211-5463.13557 PMC9989930

[CR5] Chiu DT (2001) A microfluidics platform for cell fusion. Curr Opin Chem Biol 5:609–612 11578937 10.1016/s1367-5931(00)00242-8

[CR6] de Gruijl TD, van den Eertwegh AJM, Pinedo HM, Scheper RJ (2008) Whole-cell cancer vaccination: from autologous to allogeneic tumor- and dendritic cell-based vaccines. Cancer Immunol Immunother 57:1569–1577. 10.1007/s00262-008-0536-z 18523771 10.1007/s00262-008-0536-z PMC2491427

[CR7] Dinic J, Ashrafzadeh P, Parmryd I (2013) Actin filaments attachment at the plasma membrane in live cells cause the formation of ordered lipid domains. Biochim Biophys Acta BBA - Biomembr 1828:1102–1111. 10.1016/j.bbamem.2012.12.004 10.1016/j.bbamem.2012.12.004 23246974

[CR8] Edele F, Dudda JC, Bachtanian E, Jakob T, Pircher H, Martin SF (2014) Efficiency of dendritic cell vaccination against B16 melanoma depends on the immunization route. PLoS ONE 9:e105266. 10.1371/journal.pone.0105266 25121970 10.1371/journal.pone.0105266 PMC4133283

[CR9] Fajardo-Moser M, Berzel S, Moll H (2008) Mechanisms of dendritic cell-based vaccination against infection. Int J Med Microbiol Special Issue: New Vaccination Strategies 298:11–20. 10.1016/j.ijmm.2007.07.003 10.1016/j.ijmm.2007.07.003 17719274

[CR10] Gabriel B, Teissie J (1994) Generation of reactive-oxygen species induced by electropermeabilization of Chinese hamster ovary cells and their consequence on cell viability. Eur J Biochem 223:25–33. 10.1111/j.1432-1033.1994.tb18962.x 8033899 10.1111/j.1432-1033.1994.tb18962.x

[CR11] Gabrijel M, Repnik U, Kreft M, Grilc S, Jeras M, Zorec R (2004) Quantification of cell hybridoma yields with confocal microscopy and flow cytometry. Biochem Biophys Res Commun 314:717–723. 10.1016/j.bbrc.2003.12.154 14741694 10.1016/j.bbrc.2003.12.154

[CR12] Golzio M, Teissié J, Rols M-P (2001) Control by membrane order of voltage-induced permeabilization, loading and gene transfer in mammalian cells. Bioelectrochemistry 53:25–34 11206923 10.1016/s0302-4598(00)00091-x

[CR13] Golzio M, Teissié J, Rols M-P (2002) Direct visualization at the single-cell level of electrically mediated gene delivery. Proc Natl Acad Sci 99:1292–1297 11818537 10.1073/pnas.022646499 PMC122183

[CR14] Gordy JT, Luo K, Zhang H, Biragyn A, Markham RB (2016) Fusion of the dendritic cell-targeting chemokine MIP3α to melanoma antigen Gp100 in a therapeutic DNA vaccine significantly enhances immunogenicity and survival in a mouse melanoma model. J Immunother Cancer. 10.1186/s40425-016-0189-y 28018602 10.1186/s40425-016-0189-y PMC5168589

[CR16] Haberl S, Miklavčič D, Pavlin M (2010) Effect of Mg ions on efficiency of gene electrotransfer and on cell electropermeabilization. Bioelectrochemistry 79:265–271. 10.1016/j.bioelechem.2010.04.001 20580903 10.1016/j.bioelechem.2010.04.001

[CR17] Haberl S, Kandušer M, Flisar K, Hodžić D, Bregar VB, Miklavčič D, Escoffre J-M, Rols M-P, Pavlin M (2013) Effect of different parameters used for *in vitro* gene electrotransfer on gene expression efficiency, cell viability and visualization of plasmid DNA at the membrane level: Gene electrotransfer and DNA - membrane. J Gene Med 15:169–181. 10.1002/jgm.2706 23564663 10.1002/jgm.2706

[CR18] Hawlina S, Chowdhury HH, Smrkolj T, Zorec R (2022) Dendritic cell-based vaccine prolongs survival and time to next therapy independently of the vaccine cell number. Biol Direct 17:5. 10.1186/s13062-022-00318-w 35197090 10.1186/s13062-022-00318-w PMC8864901

[CR19] Hawlina S, Zorec R, Chowdhury HH (2023) Potential of personalized dendritic cell-based immunohybridoma vaccines to treat prostate cancer. Life 13:1498. 10.3390/life13071498 37511873 10.3390/life13071498 PMC10382052

[CR20] He W, Huang L, Feng Y, Liang F, Ding W, Wang W (2019) Highly integrated microfluidic device for cell pairing, fusion and culture. Biomicrofluidics 13:054109. 10.1063/1.5124705 31893009 10.1063/1.5124705 PMC6932852

[CR21] Higano CS, Small EJ, Schellhammer P, Yasothan U, Gubernick S, Kirkpatrick P, Kantoff PW (2010) Sipuleucel-T. Nat Rev Drug Discov 9:513–514. 10.1038/nrd3220 20592741 10.1038/nrd3220

[CR22] Kanduser M, Sentjurc M, Miklavcic D (2006) Cell membrane fluidity related to electroporation and resealing. Eur Biophys J Biophys Lett 35:196–204. 10.1007/s00249-005-0021-y 10.1007/s00249-005-0021-y 16215750

[CR23] Kanduser M, Sentjurc M, Miklavcic D (2008) The temperature effect during pulse application on cell membrane fluidity and permeabilization. Bioelectrochemistry 74:52–57. 10.1016/j.bioelechem.2008.04.012 18502189 10.1016/j.bioelechem.2008.04.012

[CR24] Kanduser M, Miklavcic D, Pavlin M (2009) Mechanisms involved in gene electrotransfer using high- and low-voltage pulses—An in vitro study. Bioelectrochemistry 74:265–271. 10.1016/j.bioelechem.2008.09.002 18930698 10.1016/j.bioelechem.2008.09.002

[CR25] Kanduser M, Kokalj Imsirovic M, Usaj M (2019) The effect of lipid antioxidant α-tocopherol on cell viability and electrofusion yield of B16–F1 cells in vitro. J Membr Biol 252:105–114. 10.1007/s00232-019-00059-4 30671620 10.1007/s00232-019-00059-4

[CR26] Kandušer M, Ušaj M (2014) Cell electrofusion: past and future perspectives for antibody production and cancer cell vaccines. Expert Opin Drug Deliv 11:1885–1898. 10.1517/17425247.2014.938632 25010248 10.1517/17425247.2014.938632

[CR27] Kandušer M, Miklavčič D, Pavlin M (2009) Mechanisms involved in gene electrotransfer using high- and low-voltage pulses—An in vitro study. Bioelectrochemistry 74:265–271. 10.1016/j.bioelechem.2008.09.002 18930698 10.1016/j.bioelechem.2008.09.002

[CR28] Keenan BP, Jaffee EM (2012) Whole cell vaccines—past progress and future strategies. Semin Oncol 39:276–286. 10.1053/j.seminoncol.2012.02.007 22595050 10.1053/j.seminoncol.2012.02.007 PMC3356993

[CR29] Kim HB, Lee S, Chung JH, Kim SN, Sung CK, Baik KY (2020) Effects of actin cytoskeleton disruption on electroporation in vitro. Appl Biochem Biotechnol 191:1545–1561. 10.1007/s12010-020-03271-4 32157625 10.1007/s12010-020-03271-4

[CR30] Koido S (2016) Dendritic-tumor fusion cell-based cancer vaccines. Int J Mol Sci 17:828. 10.3390/ijms17060828 27240347 10.3390/ijms17060828 PMC4926362

[CR31] Kotnik T, Rems L, Tarek M, Miklavčič D (2019) Membrane electroporation and electropermeabilization: mechanisms and models. Annu Rev Biophys 48:63–91. 10.1146/annurev-biophys-052118-115451 30786231 10.1146/annurev-biophys-052118-115451

[CR32] Koundouros N, Poulogiannis G (2020) Reprogramming of fatty acid metabolism in cancer. Br J Cancer 122:4–22. 10.1038/s41416-019-0650-z 31819192 10.1038/s41416-019-0650-z PMC6964678

[CR33] Kozlovsky Y, Kozlov MM (2002) Stalk model of membrane fusion: solution of energy crisis. Biophys J 82:882–895 11806930 10.1016/S0006-3495(02)75450-7 PMC1301897

[CR34] Laureano RS, Sprooten J, Vanmeerbeerk I, Borras DM, Govaerts J, Naulaerts S, Berneman ZN, Beuselinck B, Bol KF, Borst J, Coosemans, an, Datsi, A., Fučíková, J., Kinget, L., Neyns, B., Schreibelt, G., Smits, E., Sorg, R.V., Spisek, R., Thielemans, K., Tuyaerts, S., De Vleeschouwer, S., de Vries, I.J.M., Xiao, Y., Garg, A.D., (2022) Trial watch: Dendritic cell (DC)-based immunotherapy for cancer. OncoImmunology 11:2096363. 10.1080/2162402X.2022.2096363 35800158 10.1080/2162402X.2022.2096363 PMC9255073

[CR35] Lee WT (2011) Dendritic cell-tumor cell fusion vaccines. Adv Exp Med Biol 713:177–186. 10.1007/978-94-007-0763-4_11 21432020 10.1007/978-94-007-0763-4_11

[CR36] Levental KR, Malmberg E, Symons JL, Fan Y-Y, Chapkin RS, Ernst R, Levental I (2020) Lipidomic and biophysical homeostasis of mammalian membranes counteracts dietary lipid perturbations to maintain cellular fitness. Nat Commun 11:1339. 10.1038/s41467-020-15203-1 32165635 10.1038/s41467-020-15203-1 PMC7067841

[CR37] Lin MJ, Svensson-Arvelund J, Lubitz GS, Marabelle A, Melero I, Brown BD, Brody JD (2022) Cancer vaccines: the next immunotherapy frontier. Nat Cancer 3:911–926. 10.1038/s43018-022-00418-6 35999309 10.1038/s43018-022-00418-6

[CR38] Lu X, Kang Y (2009) Cell fusion as a hidden force in tumor progression. Cancer Res 69:8536–8539. 10.1158/0008-5472.CAN-09-2159 19887616 10.1158/0008-5472.CAN-09-2159 PMC2783941

[CR39] Lu Y-T, Pendharkar GP, Lu C-H, Chang C-M, Liu C-H (2015) A microfluidic approach towards hybridoma generation for cancer immunotherapy. Oncotarget 6:38764–38776 26462149 10.18632/oncotarget.5550 PMC4770735

[CR40] Mac Keon S, Ruiz MS, Gazzaniga S, Wainstok R (2015) Dendritic cell-based vaccination in cancer: therapeutic implications emerging from murine models. Front Immunol. 10.3389/fimmu.2015.00243 26042126 10.3389/fimmu.2015.00243 PMC4438595

[CR41] Maccarrone M, Bladergroen M, Rosato N, Agro A (1995) Role of lipid-peroxidation in electroporation-induced cell-permeability. Biochem Biophys Res Commun 209:417–425. 10.1006/bbrc.1995.1519 7733908 10.1006/bbrc.1995.1519

[CR42] Marjanovič I, Haberl S, Miklavčič D, Kandušer M, Pavlin M (2010) Analysis and comparison of electrical pulse parameters for gene electrotransfer of two different cell lines. J Membr Biol 236:97–105. 10.1007/s00232-010-9282-1 20645081 10.1007/s00232-010-9282-1

[CR43] Markelc B, Tevz G, Cemazar M, Kranjc S, Lavrencak J, Zegura B, Teissie J, Sersa G (2012) Muscle gene electrotransfer is increased by the antioxidant tempol in mice. Gene Ther 19:312–320. 10.1038/gt.2011.97 21716301 10.1038/gt.2011.97 PMC3298856

[CR44] Muralidharan A, Boukany PE (2024) Electrotransfer for nucleic acid and protein delivery. Trends Biotechnol 42:780–798. 10.1016/j.tibtech.2023.11.009 38102019 10.1016/j.tibtech.2023.11.009

[CR45] Muralidharan A, Rems L, Kreutzer MT, Boukany PE (2021) Actin networks regulate the cell membrane permeability during electroporation. Biochim Biophys Acta BBA - Biomembr 1863:183468. 10.1016/j.bbamem.2020.183468 10.1016/j.bbamem.2020.183468 32882211

[CR46] Muralidharan A, Pesch GR, Hubbe H, Rems L, Nouri-Goushki M, Boukany PE (2022) Microtrap array on a chip for localized electroporation and electro-gene transfection. Bioelectrochemistry 147:108197. 10.1016/j.bioelechem.2022.108197 35810498 10.1016/j.bioelechem.2022.108197

[CR47] Ogawa F, Iinuma H, Okinaga K (2004) Dendritic cell vaccine therapy by immunization with fusion cells of interleukin-2 gene-transduced, spleen-derived dendritic cells and tumour cells. Scand J Immunol 59:432–439. 10.1111/j.0300-9475.2004.01411.x 15140052 10.1111/j.0300-9475.2004.01411.x

[CR48] Patente TA, Pinho MP, Oliveira AA, Evangelista GCM, Bergami-Santos PC, Barbuto JAM (2019) Human dendritic cells: their heterogeneity and clinical application potential in cancer immunotherapy. Front Immunol. 10.3389/fimmu.2018.03176 30719026 10.3389/fimmu.2018.03176 PMC6348254

[CR49] Pavlin M, Kanduser M (2015) New insights into the mechanisms of gene electrotransfer—experimental and theoretical analysis. Sci Rep 5:9132. 10.1038/srep09132 25778848 10.1038/srep09132 PMC5390920

[CR50] Pavlin M, Miklavčič D (2008) Theoretical and experimental analysis of conductivity, ion diffusion and molecular transport during cell electroporation — Relation between short-lived and long-lived pores. Bioelectrochemistry 74:38–46. 10.1016/j.bioelechem.2008.04.016 18499534 10.1016/j.bioelechem.2008.04.016

[CR51] Pavlin M, Kandušer M, Reberšek M, Pucihar G, Hart FX, Magjarevićcacute; R., Miklavčič, D., (2005) Effect of cell electroporation on the conductivity of a cell suspension. Biophys J 88:4378–4390. 10.1529/biophysj.104.048975 15792975 10.1529/biophysj.104.048975 PMC1305665

[CR52] Pavlin M, Leben V, Miklavčič D (2007) Electroporation in dense cell suspension—Theoretical and experimental analysis of ion diffusion and cell permeabilization. Biochim Biophys Acta BBA - Gen Subj 1770:12–23. 10.1016/j.bbagen.2006.06.014 10.1016/j.bbagen.2006.06.014 16935427

[CR53] Pavlin M, Flisar K, Kandušer M (2010) The role of electrophoresis in gene electrotransfer. J Membr Biol 236:75–79. 10.1007/s00232-010-9276-z 20640850 10.1007/s00232-010-9276-z

[CR54] Pavlin M, Škorja Milić N, Kandušer M, Pirkmajer S (2024) Importance of the electrophoresis and pulse energy for siRNA-mediated gene silencing by electroporation in differentiated primary human myotubes. Biomed Eng OnLine 23:47. 10.1186/s12938-024-01239-7 38750477 10.1186/s12938-024-01239-7 PMC11097476

[CR55] Perez CR, De Palma M (2019) Engineering dendritic cell vaccines to improve cancer immunotherapy. Nat Commun 10:5408. 10.1038/s41467-019-13368-y 31776331 10.1038/s41467-019-13368-y PMC6881351

[CR56] Raaijmakers TK, Ansems M (2018) Microenvironmental derived factors modulating dendritic cell function and vaccine efficacy: the effect of prostanoid receptor and nuclear receptor ligands. Cancer Immunol Immunother 67:1789–1796. 10.1007/s00262-018-2205-1 29998375 10.1007/s00262-018-2205-1 PMC6208817

[CR57] Reigada R (2014) Electroporation of heterogeneous lipid membranes. Biochim Biophys Acta BBA - Biomembr 1838:814–821. 10.1016/j.bbamem.2013.10.008 10.1016/j.bbamem.2013.10.008 24144543

[CR58] Rems L, Ušaj M, Kandušer M, Reberšek M, Miklavčič D, Pucihar G (2013) Cell electrofusion using nanosecond electric pulses. Sci Rep. 10.1038/srep03382 24287643 10.1038/srep03382 PMC3843160

[CR59] Rols M-P, Teissié J (1998) Electropermeabilization of mammalian cells to macromolecules: control by pulse duration. Biophys J 75:1415–1423 9726943 10.1016/S0006-3495(98)74060-3 PMC1299816

[CR60] Rols M, Dahhou F, Mishra K, Teissie J (1990) Control of electric-field induced cell-membrane permeabilization by membrane order. Biochemistry 29:2960–2966. 10.1021/bi00464a011 2337576 10.1021/bi00464a011

[CR61] Rosazza C, Escoffre J-M, Zumbusch A, Rols M-P (2011) The actin cytoskeleton has an active role in the electrotransfer of plasmid DNA in mammalian cells. Mol Ther 19:913–921. 10.1038/mt.2010.303 21343915 10.1038/mt.2010.303 PMC3098633

[CR62] Rosazza C, Buntz A, Rieß T, Wöll D, Zumbusch A, Rols M-P (2013) Intracellular tracking of single-plasmid DNA particles after delivery by electroporation. Mol Ther 21:2217–2226. 10.1038/mt.2013.182 23941812 10.1038/mt.2013.182 PMC3863794

[CR63] Rosenblatt J, Vasir B, Uhl L, Blotta S, MacNamara C, Somaiya P, Wu Z, Joyce R, Levine JD, Dombagoda D, Yuan YE, Francoeur K, Fitzgerald D, Richardson P, Weller E, Anderson K, Kufe D, Munshi N, Avigan D (2011) Vaccination with dendritic cell/tumor fusion cells results in cellular and humoral antitumor immune responses in patients with multiple myeloma. Blood 117:393–402. 10.1182/blood-2010-04-277137 21030562 10.1182/blood-2010-04-277137 PMC3031474

[CR64] Salomskaitė-Davalgienė S, Čepurnienė K, Satkauskas S, Venslauskas MS, Mir LM (2009) Extent of cell electrofusion in vitro and in vivo is cell line dependent. Anticancer Res 29:3125–3130 19661325

[CR65] Saxena M, Balan S, Roudko V, Bhardwaj N (2018) Towards superior dendritic-cell vaccines for cancer therapy. Nat Biomed Eng 2:341–346. 10.1038/s41551-018-0250-x 30116654 10.1038/s41551-018-0250-x PMC6089533

[CR66] Scuderi M, Dermol-Černe J, Amaral Da Silva C, Muralidharan A, Boukany PE, Rems L (2022) Models of electroporation and the associated transmembrane molecular transport should be revisited. Bioelectrochemistry 147:108216. 10.1016/j.bioelechem.2022.108216 35932533 10.1016/j.bioelechem.2022.108216

[CR67] Shi M, Su L, Hao S, Guo X, Xiang J (2005) Fusion hybrid of dendritic cells and engineered tumor cells expressing interleukin-12 induces type 1 immune responses against tumor. Tumori 91:531–538 16457153 10.1177/030089160509100614

[CR68] Shigematsu T, Koshiyama K, Wada S (2014) Molecular dynamics simulations of pore formation in stretched phospholipid/cholesterol bilayers. Chem Phys Lipids 183:43–49. 10.1016/j.chemphyslip.2014.05.005 24863643 10.1016/j.chemphyslip.2014.05.005

[CR69] Skelley AM, Kirak O, Suh H, Jaenisch R, Voldman J (2009) Microfluidic control of cell pairing and fusion. Nat Methods 6:147–152. 10.1038/nmeth.1290 19122668 10.1038/nmeth.1290 PMC3251011

[CR70] Sözer EB, Pocetti CF, Vernier PT (2018) Transport of charged small molecules after electropermeabilization — drift and diffusion. BMC Biophys 11:4. 10.1186/s13628-018-0044-2 29581879 10.1186/s13628-018-0044-2 PMC5861730

[CR71] Suzuki T, Fukuhara T, Tanaka M, Nakamura A, Akiyama K, Sakakibara T, Koinuma D, Kikuchi T, Tazawa R, Maemondo M, Hagiwara K, Saijo Y, Nukiwa T (2005) Vaccination of dendritic cells loaded with interleukin-12-secreting cancer cells augments in vivo antitumor immunity: characteristics of syngeneic and allogeneic antigen-presenting cell cancer hybrid cells. Clin Cancer Res 11:58–66 15671528

[CR72] Tan C, Dannull J, Nair SK, Ding E, Tyler DS, Pruitt SK, Lee WT (2013) Local secretion of IL-12 augments the therapeutic impact of dendritic cell–tumor cell fusion vaccination. J Surg Res 185:904–911. 10.1016/j.jss.2013.06.045 23891424 10.1016/j.jss.2013.06.045

[CR73] Tang X, Huang Q, Arai T, Liu X (2022) Cell pairing for biological analysis in microfluidic devices. Biomicrofluidics 16:061501. 10.1063/5.0095828 36389274 10.1063/5.0095828 PMC9646252

[CR74] Trontelj K, Rebersek M, Kanduser M, Serbec VC, Sprohar M, Miklavcic D (2008) Optimization of bulk cell electrofusion in vitro for production of human-mouse heterohybridoma cells. Bioelectrochemistry 74:124–129. 10.1016/j.bioelechem.2008.06.003 18667367 10.1016/j.bioelechem.2008.06.003

[CR75] Ušaj M, Kandušer M (2015) Modified Adherence Method (MAM) for Electrofusion of Anchorage-Dependent Cells. In: Pfannkuche K (ed) Cell Fusion. Springer, New York, New York, NY, pp 203–216 10.1007/978-1-4939-2703-6_15 25947667

[CR76] Ušaj M, Trontelj K, Hudej R, Kandušer M, Miklavčič D (2009) Cell size dynamics and viability of cells exposed to hypotonic treatment and electroporation for electrofusion optimization. Radiol Oncol. 10.2478/v10019-009-0017-9

[CR77] Ušaj M, Trontelj K, Miklavčič D, Kandušer M (2010) Cell-cell electrofusion: optimization of electric field amplitude and hypotonic treatment for mouse melanoma (B16–F1) and Chinese Hamster Ovary (CHO) cells. J Membr Biol 236:107–116. 10.1007/s00232-010-9272-3 20628737 10.1007/s00232-010-9272-3

[CR78] Usaj M, Kanduser M (2012) The systematic study of the electroporation and electrofusion of B16–F1 and CHO cells in isotonic and hypotonic buffer. J Membr Biol 245:583–590. 10.1007/s00232-012-9470-2 22843161 10.1007/s00232-012-9470-2

[CR79] Usaj M, Flisar K, Miklavcic D, Kanduser M (2013) Electrofusion of B16–F1 and CHO cells: The comparison of the pulse first and contact first protocols. Bioelectrochemistry 89:34–41. 10.1016/j.bioelechem.2012.09.001 23032299 10.1016/j.bioelechem.2012.09.001

[CR80] Velayuthan LP, Moretto L, Tågerud S, Ušaj M, Månsson A (2023) Virus-free transfection, transient expression, and purification of human cardiac myosin in mammalian muscle cells for biochemical and biophysical assays. Sci Rep 13:4101. 10.1038/s41598-023-30576-1 36907906 10.1038/s41598-023-30576-1 PMC10008826

[CR81] Wang J, Saffold S, Cao X, Krauss J, Chen W (1998) Eliciting T cell immunity against poorly immunogenic tumors by immunization with dendritic cell-tumor fusion vaccines. J Immunol Baltim Md 1950(161):5516–5524 9820528

[CR82] Wu M, Ke Q, Bi J, Li X, Huang S, Liu Z, Ge L (2022) Substantially improved electrofusion efficiency of hybridoma cells: based on the combination of nanosecond and microsecond pulses. Bioengineering 9:450. 10.3390/bioengineering9090450 36134996 10.3390/bioengineering9090450 PMC9495357

[CR83] Zimmermann U (1982) Electric field-mediated fusion and related electrical phenomena. Biochim Biophys Acta 694:227–277. 10.1016/0304-4157(82)90007-7 6758848 10.1016/0304-4157(82)90007-7

