# Supplementary material for: Feasibility Study for the Use of Gene Electrotransfer and Cell Electrofusion as a Single-Step Technique for the Generation of Activated Cancer Cell Vaccines
Source: J Membr Biol. 2024 Aug 12;257(5-6):377–89. doi: 10.1007/s00232-024-00320-5 (PMC11584437; doi:10.1007/s00232-024-00320-5)
Supplement: Supplementary file 1 — Supplementary file1 (DOCX 197 kb) [file 232_2024_320_MOESM1_ESM.docx]

**Supplementary data**

Table S1: Measurements of cell membrane fluidity (r-anisotropy) in conditions that can significantly affect gene electrotransfer (GFP) or cell electrofusion (ECF). We used two cell lines characterized in our previous work (B16F1 and CHO) that significantly differ in their GFP expression(Marjanovič et al., 2010) and cell fusion(Usaj and Kanduser, 2012). Electrofusion yield for B16F1 was determined at ITV_max_ 1.7 V which corresponds to 1.2 kV/cm for hypotonic buffer (hypo KPB) and 1.4 kV/cm for isotonicbuffer (iso KPB). For CHO the ITV_max_ was 1.8 V (Usaj and Kanduser, 2012).

| cell line / buffer | | iso KPB | hypo KPB | T (4°C) |
| --- | --- | --- | --- | --- |
| r-anisotropy | B16F1 | 0.187 ± 0.020 | 0.182 ± 0.014 | 0.228 ± 0.03 |
|  | CHO | 0.172 ± 0.016 | 0.171 ± 0,007 | 0.223 ± 0.01 |
| GFP | B16F1 | 22.83 ± 7.05 |  |  |
|  | CHO | 57.89 ± 2.00 |  |  |
| ECF | B16-F1 | 16.38 ± 8.56 | 32.01 ± 4.47 |  |
|  | CHO | 0.05 ± 0,05 | 10.20 ± 4.39 |  |

Figure S1: The effect of pulse parameters on a fraction of obtained hybrids that were efficiently transfected with a plasmid encoding GFP in isotonic KPB buffer.

The fraction was calculated as $fraction GFP hybrids = \frac{GFP hybrid}{hybrid}\times100$. Electric pulse parameters were: 4×200 µs (HV), 8×100 µs (HV) pulses, and a combination of HV (4×200 µs) and one LV_55_ pulse (100 ms) (HV+LV). The electric field amplitude for HV was 1.4 kV/cm and for LV 0.11 kV/cm for LV_55_. Bars are means of 3 independent experiments ± standard deviation.


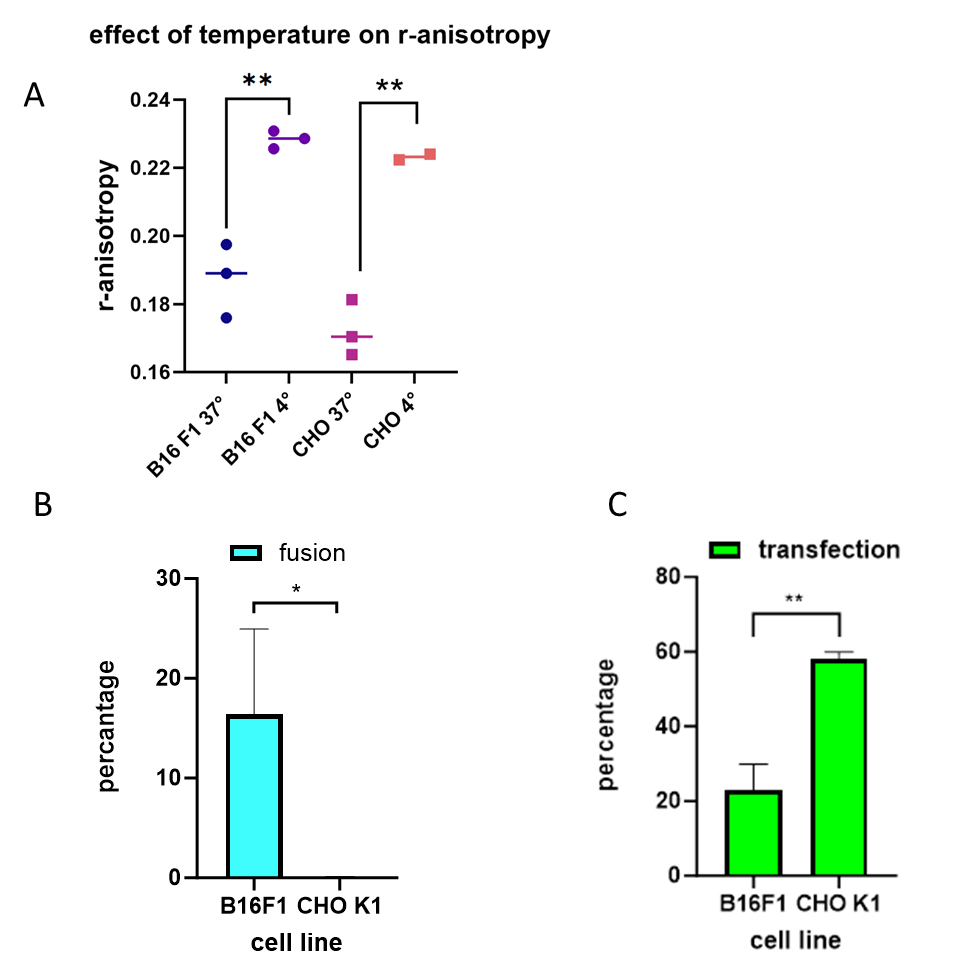


Figure S2: The effect of temperature on r-anysotropy and the percentage of cell electrofusion and gene electrotransfer in two cell lines that differ significantly in gene electrotransfer and cell electrofusion.


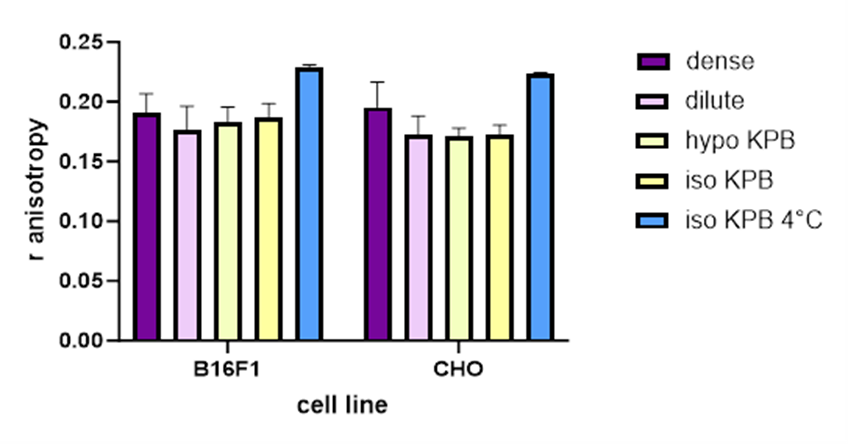


Figure S3. R-anisotropy for two different cell lines and different conditions used for gene electrotransfer or cell electrofusion protocols.
